# Reply to Sheval, E.V. Comment on “Piña et al. Ten Approaches That Improve Immunostaining: A Review of the Latest Advances for the Optimization of Immunofluorescence. *Int. J. Mol. Sci.* 2022, *23*, 1426”

**DOI:** 10.3390/ijms23084375

**Published:** 2022-04-15

**Authors:** Ricardo Piña, Isabel Acosta-Galeana, Abraham Rosas-Arellano

**Affiliations:** 1 Departamento de Biología, Facultad de Ciencias Básicas, Universidad Metropolitana de Ciencias de la Educación, Santiago 7760197, Chile; ricardo.pina@umce.cl; 2 División de Neurociencias, Instituto de Fisiología Celular, Universidad Nacional Autónoma de México, Mexico City 04510, Mexico; iacosta@ifc.unam.mx; 3 Unidad de Imagenología, Instituto de Fisiología Celular, Universidad Nacional Autónoma de México, Mexico City 04510, Mexico

We have carefully read the interesting explanatory comment by Eugene V. Sheval [1] on the review titled “Ten Approaches that Improve Immunostaining: A Review of the Latest Advances for the Optimization of Immunofluorescence” [2] published on 26 January 2022 in the *International Journal of Molecular Sciences*.

This review includes a collection of ten tips based on the experience gained at the laboratory bench of some of the authors. It also includes, as a central issue, a collection of ten summarized original proposals that had been shown to significantly improve immunofluorescence. In this section, we included a simple, rapid, and innovative method published by Svistunova and colleagues [3] entitled “A Simple Method for the Immunocytochemical Detection of Proteins Inside Nuclear Structures that are Inaccessible to Specific Antibodies”. In this study, the authors detected nucleoplasmin/B23 after a short proteinase treatment to allow immunostaining in the nucleolar structure. Proteinase treatment has been used successfully before to improve immunofluorescence methods [4,5,6]. Despite that, we decided to include Svistunova’s manuscript due to the innovative use of proteinase to locate proteins deep into the nucleolar structure.

In the methodological section of our review, we recommended methanol fixation, based on a slight confusion with the original text: the abstract section of the paper states that “In this study, the authors found that a short proteinase treatment allowed for the detection of antigens in the nucleoli”; here, fixation with formaldehyde is not mentioned. Furthermore, in the sixth paragraph of the results section, the authors wrote “Thus, using methanol fixation, it was possible to identify B23 in the nucleolar interior”. This, plus the non-significant differences observed in the average fluorescence intensity obtained with both fixation methods (formaldehyde and methanol) (Figure 2C [3]), created said confusion. Moreover, the second paragraph of the discussion affirms that “we found two ways to detect B23 in the “interior” of the nucleolus using specific antibodies: the fixation with methanol and the treatment of the fixed cells with proteases (trypsin, proteinase K, and pepsin)”. This sentence does not clearly indicate that the fixation was done using formaldehyde.

It is clear that methanol fixation alters patterns of distribution into the nucleolus, as it is stated in the original manuscript, and as we mentioned in our review; however, our principal recommendation, as shown in Figure 9 [2], is the use of trypsin with the aim of locating proteins inside nucleolar structures.

In conclusion, we appreciate the clarifying comment by Dr. Sheval for fixation with 3.7% formaldehyde as the previous step of trypsin treatment instead of methanol for the benefit of readers of the manuscript.
